# Generation and characterization of nanobodies targeting GPCR

**DOI:** 10.52601/bpr.2023.230026

**Published:** 2024-02-29

**Authors:** Shenglan Zhang, Zhiran Fan, Jianfeng Liu

**Affiliations:** 1 Bioland Laboratory (Guangzhou Regenerative Medicine and Health Guangdong Laboratory), Guangzhou 510005, China; 2 Cellular Signaling laboratory, International Research Center for Sensory Biology and Technology of MOST, Key Laboratory of Molecular Biophysics of MOE, College of Life Science and Technology, Huazhong University of Science and Technology, Wuhan 430074, China

**Keywords:** Nanobody, Single domain antibody, Heavy chain-only antibody, GPCR, G protein-coupled receptor

## Abstract

G protein-coupled receptors (GPCRs) are a large family of cell membrane proteins that are important targets for drug discovery. Nanobodies, also known as VHH (variable domains of heavy chain-only antibodies, HcAbs) antibodies, are small antibody fragments derived from camelids that have gained significant attention as potential therapeutics for targeting GPCRs due to their advantages over conventional antibodies. However, there are challenges in developing nanobodies targeting GPCRs, among which epitope accessibility is the most significant because the cell membrane partially shields the GPCR surface. We developed a universal protocol for making nanobodies targeting GPCRs using the cell membrane extract of GPCR-overexpressing HEK293 cells as the llama/alpaca immunization antigen. We constructed an immune VHH library and identified nanobodies by phage display bio-panning. The monoclonal nanobodies were recombinantly expressed in *Escherichia coli (E. coli)* and purified to characterize their binding potency.

## INTRODUCTION

G Protein-Coupled Receptors (GPCRs) are a large family of cell membrane proteins that are involved in various physiological processes. Due to their involvement in numerous diseases, GPCRs have become important targets for drug discovery (Thal *et al.* 2018). Nanobodies, also known as VHH antibodies, or single-domain antibodies, are small fragments derived from camelids such as llamas and alpacas. Nanobodies possess several advantages over conventional antibodies, including their small size, high stability, solubility, and the ability to bind with high affinity to specific target antigens (Hoey *et al.* 2019; Liu *et al.* 2018; Morrison 2019).

In recent years, nanobodies have gained significant attention as potential therapeutics, particularly in targeting GPCRs (Hutchings 2020; Jin *et al.* 2023; Muyldermans 2021). Nanobodies have emerged as powerful tools for GPCR research owing to their unique attributes, enabling researchers to stabilize GPCR conformations and providing unprecedented insights into the architecture and dynamics of these receptors. The use of nanobodies for GPCR modulation offers several advantages, including improved tissue penetration, enhanced receptor selectivity, a prolonged half-life, reduced immunogenicity, and the ability to target challenging epitopes in GPCRs (Ayoub *et al.* 2017; Jo and Jung 2016). Furthermore, nanobodies have facilitated the visualization of GPCRs through crystallography and cryo-electron microscopy, yielding high-resolution structural data that have substantially advanced our understanding of GPCR signalling mechanisms (Che *et al.* 2018, 2021; Robertson *et al.* 2022; Zhang *et al.* 2021). Due to their small size and flexibility, nanobodies can access orthosteric or allosteric sites on GPCRs, which may not be accessible to large antibodies or small molecules (De Groof *et al.* 2019; Heukers *et al.* 2019; Sheridan 2017). This property makes the nanobodies well-suited for modulating GPCR function and signalling. By targeting allosteric sites on GPCRs, nanobodies have unlocked new possibilities for modulating receptor signalling with exceptional precision, offering opportunities for the development of next-generation pharmacological agents. Moreover, engineered nanobodies have demonstrated efficacy in intracellular delivery, thereby enabling the selective manipulation of GPCR-mediated pathways and presenting prospects for innovative drug delivery modalities (Heukers *et al.* 2019; Raynaud *et al.* 2022).

The challenges in developing nanobodies targeting GPCRs are as follows: (1) Epitope Accessibility. GPCR surfaces can be partially shielded by the cell membrane, limiting the availability of accessible epitopes for nanobody binding. Identifying and designing nanobodies that bind to exposed regions or induce conformational changes to reveal orthosteric or allosteric epitopes is a significant challenge (McMahon *et al.* 2020). Typically, a purified protein is required as an antigen to generate antibodies. However, purifying GPCR in a homogeneous state *in vitro* is difficult. (2) Structural Complexity. GPCRs often adopt multiple conformational states, making it challenging to identify and optimize nanobodies to selectively target specific conformations relevant to desired therapeutic effects (Ma *et al.* 2020). (3) Conformational Flexibility. GPCRs undergo conformational changes upon ligand binding that can affect their interactions with nanobodies (Uchanski *et al.* 2020; Zimmermann *et al.* 2018). Engineering nanobodies that recognize and stabilize the desired receptor conformation is essential while avoiding undesired interactions with other states.

We focused in this protocol on the generation and characterization of nanobodies targeting GPCR, particularly the preparation of cell membrane extracts overexpressing GPCR as the appropriate antigen. We designed a universal protocol containing all procedures, from antigen preparation to nanobody characterization.

## PROTOCOL OVERVIEW

**Figure 1:**
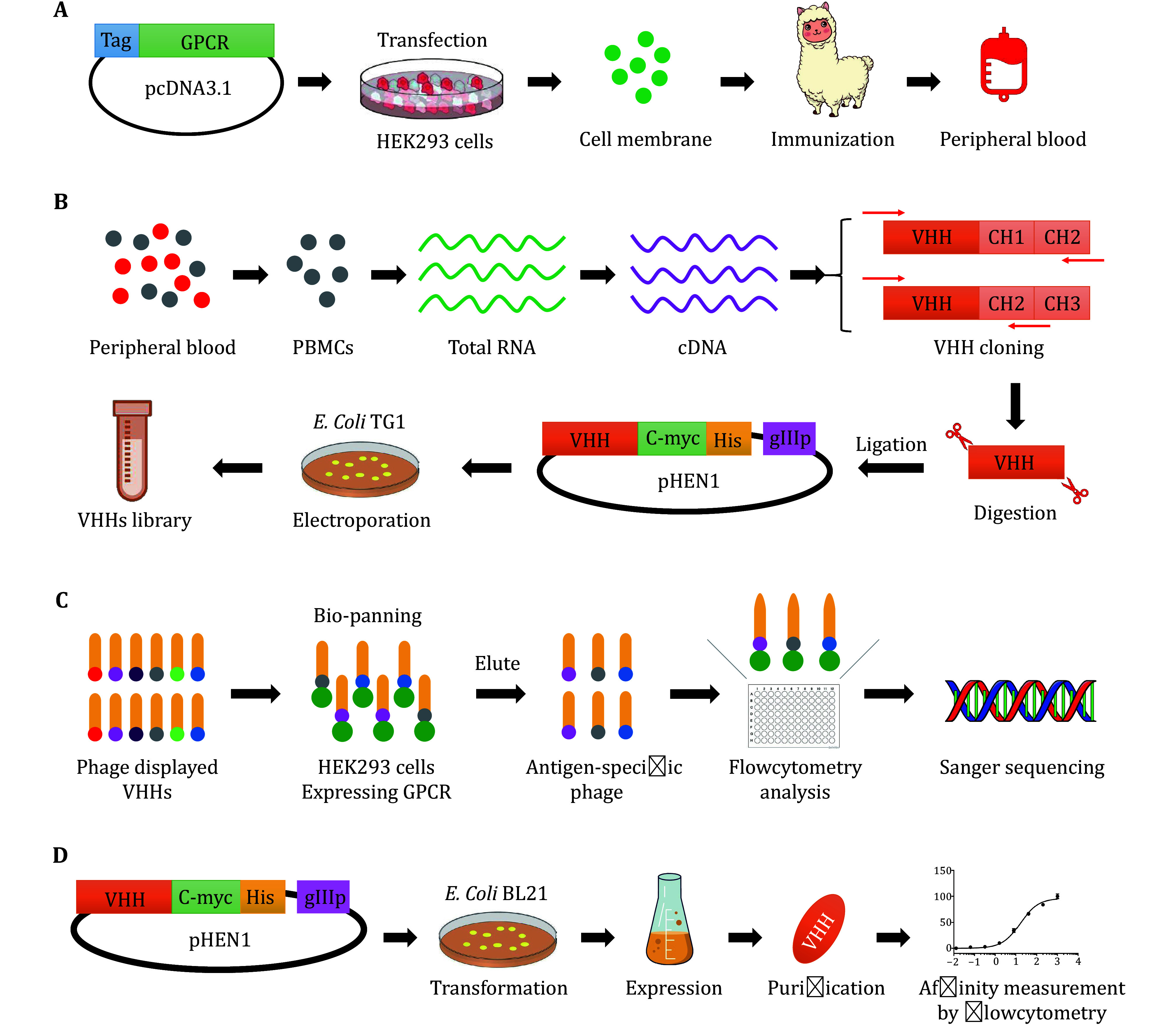
Workflow of generation and characterization of nanobodies targeting GPCR. **A** Antigen preparation and llama/alpaca immunization. **B** Immune VHH library construction. **C** Phage display selection, bio-panning and screening for nanobodies targeting GPCRs. **D** Recombinant expression, purification and affinity measurement of nanobodies

As shown in Fig.1, the gene encoding the full-length GPCR was synthesized and cloned into the mammalian cell expression vector pcDNA3.1. HEK293 cells were transfected with the plasmid, and the target GPCR product was overexpressed 1–2 days after transfection. The HEK293 cells were harvested, pelleted, and disrupted. The cell membrane containing the overexpressed GPCR was used as an antigen. To obtain nanobodies targeting GPCR, a lama/alpacas was immunized 4–6 times at 7–15 days intervals with a solution of cell membrane extraction. Peripheral blood was collected during and after immunization. The peripheral blood mononuclear cells (PBMCs) were isolated and combined (Fig. 1A). Total RNA was extracted from the PBMCs and reverse-transcribed into cDNA. VHH genes were amplified from cDNA by a two-step nested PCR, digested with restriction enzymes, and ligated into the pHEN1 phagemid vector, followed by a c-myc-tag, 6 × His-tag, an amber codon (Oh *et al.* 2007) and gene III protein (gIIIp) of the phage. The pelB signal sequence is located at the N-terminus of the VHH gene and leads to the periplasmic expression of nanobodies. The ligated phagemids were transformed into *E. coli* TG1 competent cells. All transformants were collected and stored in an immune VHH library (Fig. 1B). Dozens of colonies from the library were randomly chosen for colony PCR and Sanger sequencing analysis to evaluate the success rate of VHH gene insertion into the phagemid vector and the VHH sequence diversity of the immune library.

An immune VHH library was used to select and screen nanobodies targeting GPCR. A representative fraction of the VHH library was cultured and rescued using the KM13 helper phage to display nanobodies at the tips of the M13 phage. Phages were extracted from the *E. coli* TG1 strain and used for phage display biopanning in HEK293 cells overexpressing the target GPCR. During the bio-panning process, negative phage-displayed VHHs were removed by HEK293 cell depletion with mock transfection. The phage was enriched and eluted with a trypsin solution. After phage infection with *E. coli* TG1, a representative fraction of the bacteria was plated, and hundreds of colonies were randomly inoculated and cultured for positive clonal VHH screening. These gIIIp-fused VHHs were expressed by isopropyl-β-D-thio galactopyranoside (IPTG) induction, and the periplasmic extracts were screened for specific binding to GPCR using flow cytometry. The results of these selected clones were compared with those of negative and non-specific binding controls. Clones with a specific fluorescence intensity distribution of the anti-His-tag antibody were recognized as positive and subjected to Sanger sequencing (Fig. 1C). The remaining phages were rescued and amplified if the next round of phage display was required. Usually, at least two rounds of phage display biopanning are required for generating specific nanobodies targeting GPCR.

DNA sequencing results from the positive clones were translated into amino acids for analysis. All amino acid sequences were aligned, repeated or redundant sequences were removed, and only clones with unique amino acid sequences were retained for further analysis. The pHEN1 phagemids encoding unique VHH genes were extracted from the cultured *E. coli* TG1 and transformed into *E. coli* BL21(DE3) competent cells, as the amber codon stops translation before the *gIIIp* gene (Oh *et al.* 2007). High-purity soluble nanobodies were extracted from the periplasm and purified using a His-tagged protein purification protocol, followed by size-exclusion chromatography. The purified nanobodies were used in dose-response flow cytometry assays to characterize their binding potency to GPCR. Calibration curves were used to determine the affinity of the nanobodies for GPCR (Fig. 1D).

## MATERIALS

### Reagents and materials

• Complete Freund’s adjuvant: ThermoFisher Scientific, Cat. #77140

• Incomplete Freund’s adjuvant: ThermoFisher Scientific, Cat. #77145

• Accutase solution: Merk, Cat. #A6964

• Reverse Transcriptase: Sigma-Aldrich, Cat. #3531287001

• RNase inhibitor: Sigma-Aldrich, Cat. #R2520

• dNTP mix: ThermoFisher Scientific, Cat. #18427013

• Amicon UltraCentrifugal Filter Units: Merk, Cat. #UFC8010

• Trypsin solution: Sigma-Aldrich, Cat. #T1426

• AzBTS tablets: Sigma-Aldrich, Cat. #A9941

• Polyethylene glycol-8000: Sigma-Aldrich, Cat. #89510

• Mammalian Total RNA Miniprep kit: Sigma-Aldrich, Cat. #RTN350

• Plasmid Miniprep kit: Sigma-Aldrich, Cat. #NA9604

• Bugbuster Master Mix: Merk, Cat. #71456

• Restriction enzyme *NotI*: New England Biolabds, Cat. #R0189L

• Restriction enzyme *SfiI*: New England Biolabds, Cat. #R0123L

• Antartic Phosphatase: New England Biolabds, Cat. #M0289S

• T4 DNA ligatase: New England Biolabds, Cat. #M0202S

• Phusion High-Fidelity DNA Polymerase: New England Biolabds, Cat. #M0530L

• DreamTaq DNA Polymerase: ThermoFisher Scientific, Cat. #EP0702

• *E. Coli* TG1 electroporation-competent cells: Lucigen, Cat. #60502-2

• Gel and PCR clean-up kit: Macherey-Nagel, Cat. #740609.250

• Trypton: Oxoid, Cat. #LP0042B

• Yeast extract: Oxoid, Cat. #LP0021B

• Agar power: Oxoid, Cat. #LP0011B

• Bovin serum albumin (BSA): BioFroxx, Cat. #4240GR100

• Ampicillin: BioFroxx, Cat. #1146GR001

• Kanamycin: BioFroxx, Cat. #1162GR025

• Isopropyl-β-D-thiogalactopyranoside (IPTG): BioFroxx, Cat. #1122GR1000

• 96-Well Polystyrene Conical Bottom MicroWell Plates (V-bottom): ThermoFisher Scientific, Cat. #249952

• His Tag Alexa Fluor 647-conjugated Antibody: R&D Systems, Cat. #IC0501R

• Ficoll-Paque PLUS density gradient medium: Cytiva, Cat. #17144003

• Cobalt-based His-tagged protein purification resin: Cytiva, Cat. # 28957502

• Superdex-75 increased size exclusion chromatography column: Cytiva, Cat. # 29148721

• NaCl: Macklin, Cat. #S805275

• Sucrose: Macklin, Cat. #S818046

• Tris: Macklin, Cat. #T818967

• EDTA: Macklin, Cat. #E809186

• EGTA solution: Macklin, Cat. #E885218

• Sucrose: Macklin, Cat. #S818049

• HEPES: Macklin, Cat. #H822240

• Imidazole: Macklin, Cat. #I6122

• Tween-20: Macklin, Cat. #T6335

• Phosphate buffered saline (PBS): ThermoFisher Scientific, Cat. #10010023

### Buffers and culture medium

• 2YT medium: 16 g Trypton, 10 g Yeast extract and 5 g NaCl in 1 L ultrapure water

• Dulbecco's Modifed Eagle Medium (DMEM): ThermoFisher Scientific, Cat. #C11995500CP

• Cell membrane buffer: 5 mmol/L EGTA, 1 mmol/L EDTA, 10% sucrose (*w*/*v*) and 50 mmol/L HEPES pH 7.5 in ultrapure water

• PEG-NaCl buffer: 800 g PEG-8000 and 146.1 g NaCl in 1 L ultrapure water

• Wash buffer: PBS containing 30 mmol/L imidazole

• Elute buffer: PBS containing 300 mmol/L imidazole

### Equipment

• Dounce tissue grinder set, Sigma-Aldrich D9188

• Refrigerated centrifuge, Eppendorf 5910R

• Refrigerated Microcentrifuge, Sorvall Legend Micro 21

• Untracentrifuge, ThermoFisher Scientific Sorvall WX 100+

• PCR, Bio-Gener RePure

• Microplate reader: TECAN, Infinite M nano

• ThermoCell MixingBlock: Bioer MB-102

• Gel electrophoresis system, Tanon HE220 and EPS200

• Gel imaging system, Tanon 1600

• MicroPulser Electroporator, Bio-Rad

• Shaking incubator, Minquan MQT-60R

• Biochemical incubator, Yiheng LRH-150

• Protein purification system, Cytiva AKTA pure 25L

• Flow cytometry, Angilent Novocyte Advanteon

### Software

• NovoExpress v1.5.0, Angilent Technologies, Inc.

• Graphpad Prism v8.0, GraphPad Software, LLC

• BioEdit v7.0.9 (RRID: SCR_007361)

• MAFFT v7.490 (Katoh *et al.* 2002)

• ProtParam (Wilkins *et al.* 1999)

## PROCEDURES

### Step 1: Antigen preparation

Step 1.1: The synthetic gene encoding the full-length target GPCR was cloned into a mammalian expression vector (pcDNA3.1) containing an HA or FLAG-tag at the C-terminus for the GPCR expression test.

Step 1.2: Transfect HEK293 cells with the GPCR expression plasmid for 1–2 days. At least 2 × 10^8^ cells should be prepared.

Step 1.3: Harvest all the transfected HEK293 cells with Accutase solution.

Step 1.4: Pellet the HEK293 cells expressing the target GPCR by refrigerated centrifugation. In order to avoid internalization of the target GPCR and protein degradation by proteases, it is essential to keep the proteins at 4 °C throughout the protocol.

Step 1.5: Resuspend the cell pellet in 10 mL of the cell membrane buffer and homogenize the cells with a Dounce tissue grinder for 5 min.

Step 1.6: Centrifuge the cells for 10 min at 1000 *g* to pellet the cell debris.

Step 1.7: Keep the supernatant and repeat Steps 1.5 and 1.6 2–3 times.

Step 1.8: Combine all the supernatants and ultracentrifuge for 30 min at 100,000 *g*.

Step 1.9: Resuspend the pellet in 5 mL of PBS and store at –80 °C.

**[CRITICAL]** Using the anti-HA- or FLAG-tag antibody, it is important to check the expression level of target GPCR on the HEK293 cell surface by immunoassays (*e*.*g*., flow cytometry or cell-based ELISA).

### Step 2: Llama/alpaca immunization

Step 2.1: Thaw the HEK293 cell membrane suspension and divide it into 4–6 equal parts in the vials, indicating the immunization times (4–6).

Step 2.2: Mix the cell membrane suspension in an equal volume of Freund’s complement (for the first immunization) or complement adjuvant as the immunogens.

Step 2.3: Immunize a healthy young llama or alpaca subcutaneously 4–6 times at 7–15 days intervals.

Step 2.4: On the day before and 14 days after the last immunization, collect approximately 200 mL of peripheral blood from the llama and alpaca.

Step 2.5: Isolate the PBMCs using Ficoll-Paque PLUS density gradient medium and combine the PMBCs.

### Step 3: VHH library construction

Step 3.1: Extract the total RNA from the PBMCs using the Mammalian Total RNA Miniprep kit.

Step 3.2: Synthesize the cDNA using the total RNA as the template. Use RNAse inhibitors to avoid RNA degradation.

Step 3.3: Use the forward primer CH2FORTA4 (Arbabi Ghahroudi *et al.* 1997), an equimolar mixture of the four reverse primers (Table 1) (Behar *et al.* 2009), and the synthesized cDNA as the template to perform the first PCR using Phusion High-Fidelity DNA Polymerase.

**Table 1:** Primers used for the VHH library construction

Primers	Sequence (5’ – 3’)
CH2FORTA4	CGCCATCAAGGTACCAGTTGA
5′ VH1–Sfi	CATGCCATGACTCGCGGCCCAGCCGGCCATGGCCCAGGTGCAGCTGGTGCAGTCTGG
5′ VH2–Sfi	CATGCCATGACTCGCGGCCCAGCCGGCCATGGCCCAGGTCACCTTGAAGGAGTCTGG
5′ VH3–Sfi	CATGCCATGACTCGCGGCCCAGCCGGCCATGGCCGAGGTGCAGCTGGTGGAGTCTGG
5′ VH4–Sfi	CATGCCATGACTCGCGGCCCAGCCGGCCATGGCCCAGGTGCAGCTGCAGGAGTCGGG
3’ VHH-Not	CCACGATTCTGCGGCCGCTGAGGAGACRGTGACCTGGGTCC

Step 3.4: Identify the amplification of two bands corresponding to the VH-CH1 hinge and part of the CH2 gene fragment of traditional antibodies (approximately 1000 bp) or the VHH hinge and part of the CH2 gene fragment of HcAbs (approximately 800 bp) using agarose gel electrophoresis (1.5 % agarose, *w*/*v*).

Step 3.5: Purify the lower band (VHH hinge and part of the CH2 gene) using a gel and PCR clean-up kit.

Step 3.6: Use the forward primer 3′ VHH-Not (Table 1)**,** and an equimolar mixture of the four backward primers containing *SfiI* and *NotI* restriction enzyme sites and purified VHH-CH2 as the template to perform the second PCR via DreamTaq DNA Polymerase.

Step 3.7: Identify the amplification of the VHH gene (approximately 400 bp) by agarose gel electrophoresis (1.5 % agarose, *w*/*v*).

Step 3.8: Purify the VHH gene using a gel and PCR clean-up kit.

Step 3.9: Digest the purified VHH gene with *SfiI* and *NotI* restriction enzymes, then purify the digested VHH gene as the “Inserts” using a gel and PCR clean-up kit.

Step 3.10: Digest the pHEN1 phagemid vector with *SfiI* and *NotI* restriction enzymes, then purify the digested vector as the “Vectors” using a gel and PCR clean-up kit.

Step 3.11: Ligate the “Inserts” into “Vectors” using T4 DNA ligase. At least 5 µg of “Inserts” should be used to prepare a large VHH library; the amount of “Vectors” used for ligation must be tested.

Step 3.12 Transform all the ligated materials into TG1 *E. coli* electroporation-competent cells and coat the bacteria in the 2YT agar medium with ampicillin (0.1 mg/mL) and 2 % glucose (*m*/*v*) at 37 °C to grow.

Step 3.13 Harvest all the grown colonies and centrifuge for 15 min at 3000 *g*.

Step 3.14 Resuspend the bacteria pellet in 3–5 mL of PBS containing 20 % glycerol (*v*/*v*) as the immune VHH library and store it at –80 °C.

**[CRITICAL]** The quality of the library is reflected in both the capacity and transformation quality, where the capacity is assessed by counting the number of colonies grown on agar plates, and the positive rate of colony PCR identifies the transformation quality.

### Step 4: Phage display selection and bio-panning

Step 4.1: Grow 50 µL of the VHH library in 50 mL of 2YT medium with ampicillin (0.1 mg/mL) and 2 % glucose (*m*/*v*) at 37 °C with shaking to an OD_600_ around 0.6.

Step 4.2: Infect the bacteria using the KM13 helper phage and incubate at 37 °C for 30 min.

Step 4.3: Centrifuge for 15 min at 3000 *g* to pellet the bacteria.

Step 4.4: Resuspend the pellet in 250 mL of 2YT medium with ampicillin (0.1 mg/mL) and kanamycin (0.05 mg/mL) overnight at 30 °C with shaking.

Step 4.5: Culture the bacteria overnight, split into ten vials, and centrifuge for 30 min at 3000 *g*.

Step 4.6: Add 5 mL (1/5 volume of bacteria in each vial) of PEG-NaCl buffer to the supernatant in a new vial and incubate on ice for 1 h.

Step 4.7: Centrifuge for 30 min at 3000 *g*, and resuspend the phage pellet in 1 mL of PBS.

Step 4.8: Centrifuge for 5 min at 16,000 *g* to remove the bacterial contaminants.

Step 4.9: Add 0.2 mL of PEG-NaCl buffer to the supernatants in a new vial and incubate on ice for 30 min.

Step 4.10: Centrifuge for 5 min at 16,000 *g* to pellet the phage.

Step 4.11: Resuspend the pellet in 1 mL of PBS as the phage library.

Step 4.12: Before phage display and biopanning, transfect HEK293 cells for 1–2 days with the plasmid encoding the target GPCR. Prepare HEK293 cells using mock transfection.

Step 4.13: Harvest the HEK293 cells with GPCR or mock transfection using the Accutase solution.

Step 4.14: Saturate the phage library and HEK293 cells with PBS-2% BSA (*w*/*v*) at 4 °C for 1 h.

Step 4.15: Combine the phage and HEK293 cells with mock transfection, incubating at 4 °C for 1 h with shaking as the depletion for non-specific binders.

Step 4.16: Centrifuge for 5 min at 1000 *g* to pellet the cells.

Step 4.17: Transfer the supernatant (depleted phage) into HEK293 cells overexpressing target GPCR and incubate at 4 °C for 2 h with shaking.

Step 4.18: Wash the cells three times with 1 mL of PBS containing 0.1% tween-20 (*v*/*v*) and three times with PBS to remove non-specific binding.

Step 4.19: Elute the bound phages using a 1-mg/mL trypsin solution for 20 min at room temperature with shaking.

Step 4.20: Rescue the phage in the *E. coli* TG1 strain; plate a part of the infected bacteria on 2YT agar medium with ampicillin (0.1 mg/mL) and 2% glucose (*m*/*v*) and keep at 37 °C overnight.

### Step 5: Screening for GPCR-specific nanobodies

Screen individual *E. coli* TG1 colonies from the phage display selection using flow cytometry.

Step 5.1: Place the colonies randomly into different 96-deep-well plates in 400 μL of 2YT medium with ampicillin (0.1 mg/mL) and 2% glucose (*m*/*v*) at 37 °C for 4 h with shaking. (NOTE: Each plate contained a negative control (no colonies) and a non-specific control preserved in our laboratory (a colony encoding a VHH gene that binds to a non-specific target on the surface of HEK293 cells)).

Step 5.2: Induce the grown bacteria to produce nanobodies by IPTG at 30 °C overnight.

Step 5.3: Before phage display and bio-panning, transfect HEK293 cells for 1–2 days with the plasmid encoding the target GPCR.

Step 5.4: Harvest the HEK293 cells using accutase solution and split them into 96-well Polystyrene Conical Bottom MicroWell Plates (V-bottom) — at least 10^5^ cells/well.

Step 5.5: Saturate the cells with PBS-2% BSA (*w*/*v*) at room temperature for 1 h.

Step 5.6: Centrifuge for 30 min at 3000 *g* to pellet the bacteria in the 96-deep-well plate and lyse by freeze-thawing with Bugbuster Master Mix solution (0.1 mL/well).

Step 5.7: Add 50 µL of the bacterial supernatant from different 96-deep-well plates into the 96-well Polystyrene Conical Bottom MicroWell Plates and incubate at room temperature for 2 h with shaking.

Step 5.8: Wash the cells three times with 1 mL of PBS containing 0.1% tween-20 (*v*/*v*) and three times with PBS to remove non-specific binding.

Step 5.9: Add 10 µL of His Tag Alexa Fluor 647-conjugated antibody (1:5000 dilution in PBS) to the 96-well Polystyrene Conical Bottom MicroWell Plates, 0.05 mL/well, and shake for 2 h at room temperature.

Step 5.10: Wash the cells three times with 1 mL of PBS containing 0.1% tween-20 (*v*/*v*) and three times with PBS to remove non-specific binding.

Step 5.11: Resuspend the cells in PBS (100 µL/well) and carry out flow cytometry assays.

Step 5.12: Compare the results with the negative and non-specific controls.

Step 5.13: Keep the positive colonies and send them for Sanger sequencing.

Step 5.14: Translate the DNA sequences into amino acids and align all nanobody sequences using MAFFT software. Perform sequence alignment, remove the repeated sequences, and retain unique VHH colonies for further analysis.

### Step 6: Expression and purification of nanobodies

Step 6.1: Prepare the phagemids of positive colonies from the screening step using the Plasmid Miniprep kit.

Step 6.2: Transform phagemids into *E. coli* BL21(DE3) competent cells for large-scale nanobody production. Grow transformed bacteria in 200 mL of 2YT medium with ampicillin (0.1 mg/mL) at 37 °C with shaking until OD_600_ = 0.8; then, induce bacteria with 0.1 mmol/L IPTG for overnight growth at 30 °C with shaking.

Step 6.3: Centrifuge for 30 min at 3000 *g* to pellet the bacteria.

Step 6.4: Resuspend the bacteria in 10 mL of Bugbuster mix solution and incubate for 30 min with shaking.

Step 6.5: Centrifuge for 30 min at 20,000 *g* to pellet the debris.

Step 6.6: Transfer the supernatant into a new vial with PBS-washed cobalt-based His-tagged protein purification resin and incubate for 1 h with shaking.

Step 6.7: Wash the resin three times with a wash buffer.

Step 6.9: Elute the resin five times with elute buffer.

Step 6.10: Concentrate the eluted proteins containing nanobody to 0.5–1 mL using Amicon UltraCentrifugal Filter Units.

Step 6.11: Perform size exclusion chromatography in PBS using Superdex 75 increased column supplemented with an AKTA pure 25L protein purification system.

Step 6.12: Concentrate the purified nanobody using Amicon UltraCentrifugal Filter Units. Determine the protein concentration of the nanobody with a microplate reader using the molar extinction coefficient calculated by the online program ProtParam (Wilkins *et al.* 1999).

### Step 7: Affinity measurement of GPCR-specific nanobodies

The binding potency of purified nanobodies is measured by their affinity using flow cytometry.

Step 7.1: Before affinity measurement, transfect HEK293 cells for 1–2 days with the plasmid encoding the target GPCR.

Step 7.2: Harvest the HEK293 cells using Accutase solution and split them into 96-well Polystyrene Conical Bottom MicroWell plates — at least 10^5^ cells/well.

Step 7.3: Saturate the cells with PBS-2% BSA (*w*/*v*) at room temperature for 1 h.

Step 7.4: Centrifuge for 10 min at 1000 *g* to pellet cells.

Step 7.5: Prepare nanobody solutions at concentrations of 5000, 1000, 200, 40, 8, 1.6, 0.32, and 0.064 nmol/L. Add the nanobody solutions to cells in 96-well Polystyrene Conical Bottom MicroWell Plates and incubate for 2 h with shaking.

Step 7.6: Centrifuge for 10 min at 1000 *g* to obtain a pellet. Wash the cells three times with 0.15 mL/well PBS containing 0.1% tween-20 (*v*/*v*) and three times with PBS to remove non-specific binding.

Step 7.7: Add 10 µL of His Tag Alexa Fluor 647-conjugated antibody (1:5000 dilution in PBS) to 96-Well Polystyrene Conical Bottom MicroWell Plates, 0.05 mL/well, and keep for 2 h at room temperature with shaking.

Step 7.8: Centrifuge for 10 min at 1000 *g* to obtain a pellet. Wash the cells three times with 0.15 mL/well PBS containing 0.1% tween-20 (*v*/*v*) and three times with PBS to remove non-specific binding.

Step 7.9: Resuspend the cells in PBS (100 µL/well) and carry out flow cytometry assays.

Step 7.10: Analyze the results using the median or mean fluorescence intensity as the statistical value to characterize the binding capacity. Fit the curves (the *x*-axis indicating concentrations of nanobodies and the *y*-axis the statistical values) using GraphPad Prism software by the nonlinear dose-response method to determine the affinity of the nanobodies, which was represented by the equilibrium dissociation constant (KD) between the nanobody and GPCR, using a one-site specific binding equation:

$  Y={B}_{\max}\times X/(KD + X) , $

where *X* is the concentration of nanobodies, *Y* is the response to the specific binding, *B*_max_ is the maximum specific binding in the same units as *Y*, and *KD* is the equilibrium dissociation constant in the same units as *X*.

## CONCLUSION AND DISCUSSION

We describe this protocol as a universal and unbiased workflow for generating and characterizing nanobodies targeting GPCR. Using membrane extraction of HEK293 cells expressing GPCR instead of purified proteins, which is the traditional protocol of antibody development, greatly improves the success rate of nanobody generation, leading to nanobodies that recognize the native epitopes of GPCR. Further, more focused strategies could be optimized based on our universal protocol, such as adding a GPCR ligand to stabilize the conformation during phage display biopanning and using genetic mutants during antigen preparation. We hope this protocol will contribute to the research and development of nanobodies.

## Conflict of interest

Shenglan Zhang, Zhiran Fan and Jianfeng Liu declare that they have no conflict of interest.
